# The Effect of Air Turbulence on Vortex Beams in Nonlinear Propagation

**DOI:** 10.3390/s23041772

**Published:** 2023-02-04

**Authors:** Di Zhu, Chunhua Li, Xiaodong Sun, Yali Liu, Yuqi Zhang, Hui Gao

**Affiliations:** 1 School of Electronics and Information Engineering, Tiangong University, Tianjin 300387, China; 2 Tianjin Key Laboratory of Optoelectronic Detection Technology and Systems, Tianjin 300387, China; 3 School of Physical Science and Technology, Tiangong University, Tianjin 300387, China

**Keywords:** filamentation, turbulence, vortex beam, axicon

## Abstract

Vortex beams with orthogonality can be widely used in atmospheric applications. We numerically analyzed the statistical regularities of vortex beams propagating through a lens or an axicon with different series of turbulent air phase screens. The simulative results revealed that the distortion of the transverse intensity was sensitive to the location and the structure constant of the turbulence screen. In addition, the axicon can be regarded as a very useful optical device, since it can not only suppress the turbulence but also maintain a stable beam pattern. We further confirmed that a vortex beam with a large topological charge can suppress the influence of air turbulence. Our outcomes are valuable for many applications in the atmospheric air, especially for optical communication and remote sensing.

## 1. Introduction

Ultrafast laser filamentation has attracted more and more interest due to its potential applications, such as THz generation [1,2], pulse compression [3,4], air lasing [5,6] and remote sensing [7,8]. Ultrafast laser filamentation is a unique nonlinear optical phenomenon that occurs in transparent optical media when the laser reaches the critical power (P_cr_ ≈ 5 GW in air) [9,10,11]. It is mainly caused by the dynamic balance between the optical Kerr-effect-induced self-focusing and defocusing effects of the plasma generated by a high-intensity laser or higher-order Kerr effect (HOKE) [12,13]. Fruitful nonlinear optical phenomena can be observed during the ultrafast laser filamentation evolution, including self-focusing, group velocity dispersion (GVD), self-steepening, self-phase modulation (SPM), multi-photon and tunneling ionization (MPI/TI) and stimulated amplification [9], etc. Recently, several studies demonstrated that ultrafast laser filamentation was able to achieve free space optical (FSO) communication through clouds and fog in the atmosphere [14,15]. These reports open a new and exciting prospect for the atmospheric application of optical communication.

As is known, a vortex beam is considered to have many promising applications since its special helical wavefront can generate the orbital angular momentum (OAM). A vortex beam is characterized by the phase factor exp(ilθ), where *θ* is the azimuthal coordinate and *l,* termed the topological charger, refers to an integer counting the number of intertwined helices, which represents the number of 2*π* phase shifts along the circle [16]. The mode of OAM state is equal to this number *l*. A vector normal to a helical phase front follows a spiral trajectory around the propagation axis. The different OAM modes of the vortex beam are orthogonal with each other [17]. More importantly, it is found that a vortex beam with orthogonality can be widely used in FSO communication and remote sensing systems [18,19]. In the past decade, several methods have been proposed by which vortex beams carrying OAM can intensively increase the information rate and capacity of FSO communication systems [20,21,22,23,24]. Although a vortex beam usually has a ring structure in transverse modulation, it still undergoes the process of self-focusing and defocusing effects when the peak intensity of the laser power is high enough to exceed the critical power. Therefore, a vortex beam carrying OAM during nonlinear propagation is also considered to be laser filamentation.

These types of atmospheric applications, such as optical communication [25,26,27,28], remote sensing and LIDAR technology (light detection and ranging) [29], etc., require the long-distance propagation of the laser beam through the atmosphere. Turbulent air is the main obstacle during the propagation of ultrafast filamentation through the atmosphere. It generates optical perturbation in the air density, resulting in fluctuations in the refractive index in the atmosphere and instabilities in the optical transverse modulation [25,26]. Some studies were devoted to the influence of turbulent air on the filament distance [30,31], optical pulse broadening [32] and transverse filament wandering [33,34], etc. Due to the existence of turbulence, the vortex beam will suffer from serious beam expansion, drift and distortion. Consequently, the code information that is carried by the vortex beam will be easily lost, and the blurring and scintillation that are generated by phase-front distortions will severely degrade the system’s stability [35,36]. Therefore, it is very significant to deeply understand the influence of turbulent air on the process of filamentation.

In this paper, we present the effect of air turbulence on the transverse beam wandering when vortex beams propagate through a lens or an axicon during nonlinear propagation. In order to obtain the statistical regularities of vortex beam propagation in turbulent air, we used 20 independent simulations with different series of stochastic turbulence phase screens for each condition. The statistical analyses found that beam wandering was affected by the location and the structure constant of the turbulence screens. Meanwhile, it was found that a nonlinear effect is better in suppressing the air turbulence during propagation. In addition, in our simulations, the axicon could be regarded as a very useful optical device in not only suppressing the turbulence, but also maintaining a stable beam pattern. We further confirmed that a large topological charge can reduce the influence of air turbulence during propagation. Our results are meaningful to remote sensing and optical communication in the atmosphere.

## 2. Numerical Simulation Model

The numerical simulation model of vortex beam propagation through air turbulence consists of two parts, including the filamentation model and the turbulent phase screen model. Our theoretical study mainly relies on the numerical simulations of a 2D + 1 [*A*(*x*,*y*,*z*)] nonlinear wave equation [33,37]:

(1) $$2ik_{0}\frac{\partial A}{\partial z}+(\frac{\partial^{2}}{\partial x^{2}}+\frac{\partial^{2}}{\partial y^{2}})A+2k_{0}^{2}\Delta nA+2k_{0}^{2}\tilde{n}(x,y,z)A=0,$$

where *A* denotes the amplitude of a continuous wave beam, and *k*_0_ represents the wave number of the beam, whose central wavelength is 800 nm. In Equation (1), Δ*n* corresponds to the intensity-dependent refractive index, which is determined by the optical Kerr effect ($\Delta n_{\ker}=n_{2}I$, *n*_2_ = 2 × 10^−19^ cm^2^/W) and the plasma defocusing effect ($\Delta n_{plasma}=-\sigma I^{m}$)-induced nonlinear refractive index. Here, *σ* is an empirical parameter determined from our previous work, which gives rise to a clamped intensity of 5 × 10^13^ W/cm^2^ [38] in air. Term *m* is chosen to be equal to 8, which is approximately the reported effective nonlinearity order in air by a near-infrared femtosecond laser [39]. Since we focus mainly on the spatial distribution of the vortex beam, the temporal aspects of the nonlinear propagation are not considered in Equation (1). The validity of this type of simplified simulation model has been clarified by previous studies on multi-filamentation [40,41].

Term $\tilde{n}(x,y,z)$ of Equation (1) corresponds to the fluctuations in the air refractive index caused by the air turbulence. Corresponding to the reality of optical perturbation in the atmosphere, we use the classical model of the modified Karman spectrum describing the stationary, isotropic and homogeneous turbulent air [34]:

(2) $$\phi_{n}(\kappa_{x},\kappa_{y},\kappa_{z})=0.033C_{n}^{2}{\left(\kappa^{2}+\kappa_{0}{}^{2}\right)}^{-11/6}\exp[-(\kappa^{2}/\kappa_{m}^{2})],0\leq \kappa <\infty ,$$

where *C_n_*^2^ denotes the refractive index structure constant, whose values vary from 10^−15^ cm^−2/3^ in relative weak air turbulence to approximately 10^−11^ cm^−2/3^ in relatively strong turbulence. Term κ refers to the spatial wave number, which is given by $\kappa =\sqrt{\kappa_{x}^{2}+\kappa_{y}^{2}+\kappa_{z}^{2}}$, while $\kappa_{m}=5.92/l_{0}$ and $\kappa_{0}=2\pi /L_{0}$. Terms $l_{0}$ and $L_{0}$ are the inner and outer scales of turbulent air, respectively. The scale of the air turbulence air refractive index fluctuations varies from the inner scale $l_{0}$, which is approximately 0.1~1 cm, to the outer scale $L_{0}$, which can be several meters [42].

In practice, the typical air turbulence phase image consists of a number of superimposed turbulence spectral densities distributed over a distance. During the propagation, we divide the cumulative modified Karman spectrum into several parts, and each part is regarded as a complete “sub-phase screen”, which has a unified amplitude. The spectral density of phase fluctuations on each part Δz has the form of

(3) $$F_{\phi }(\kappa_{x},\kappa_{y})=2\pi k_{0}^{2}\Delta z\phi_{n}(\kappa_{x},\kappa_{y},0).$$

According to the method described in previous studies [42,43], the spatial phase fluctuations are reconstructed by the fast Fourier transform (FFT) of the spatial spectrum density indicated by Equation (3). The random complex 2D field of air turbulence phase screen ϕ(x,y) is given by

(4) $$\phi (x,y)=\frac{1}{\sqrt{NM}}\sum_{p=-\frac{N}{2}}^{\frac{N}{2}-1}\sum_{q=-\frac{M}{2}}^{\frac{M}{2}-1}\alpha_{pq}(\varepsilon_{pq}+i\eta_{pq})\exp[i2\pi (\frac{px}{N}+\frac{qm}{M})]$$

where *N* and *M* are the numbers of the numerical simulation grids. Terms $\varepsilon_{pq}$ and $\eta_{pq}$ are statistically independent random numbers, and $\alpha_{pq}$ is determined by the spectral density of phase fluctuations as follows:

(5) $$\alpha_{pq}^{2}=F_{\phi }(p\Delta k_{x},q\Delta k_{y})\Delta k_{x}\Delta k_{y}$$

where $\Delta k_{x}$ and $\Delta k_{y}$ are connected with the transverse dimensions of the phase screen *L_x_* and *L_y_*, respectively, as

(6) $$\Delta k_{x}=\frac{2\pi }{L_{x}},\Delta k_{y}=\frac{2\pi }{L_{y}}$$

Finally, the air refractive index fluctuations $\tilde{n}(x,y,z)$ of Equation (1) can be expressed as follows:

(7) $$\tilde{n}(x,y,z)=\frac{\phi (x,y)}{k_{0}^{}\Delta z}.$$

**Figure 1:**
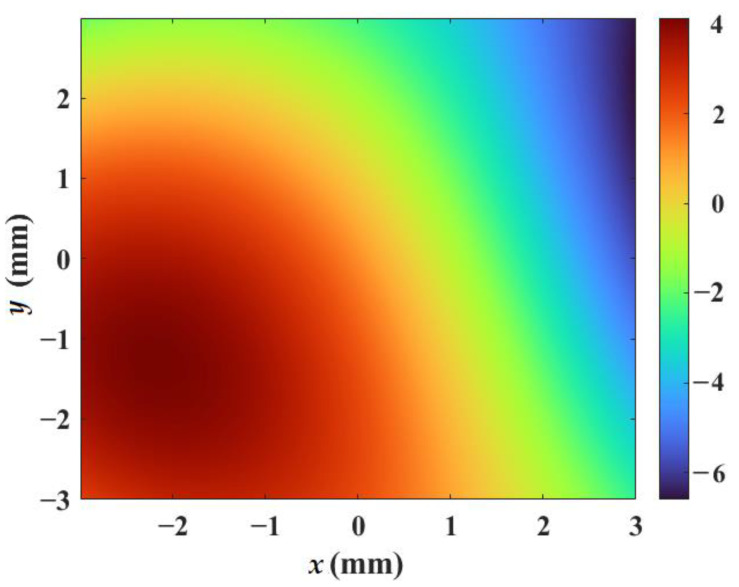
Representative turbulent phase screen with the structure constant C*_n_*^2^ = 5.6 × 10^−13^ cm^−2/3^ and the distance Δ*z* = 1 m.

We chose the numerical simulation grids (1200 × 1200) with a unit grid size of 5 μm for both the vortex beam propagation model and the turbulent phase screen model. Because of the random and stochastic characteristic, as an illustration, Figure 1 shows the representative simulated turbulent phase screen with the structure constant C*_n_*^2^ = 5.6 × 10^−13^ cm^−2/3^ and the distance Δ*z* = 1 m. The color scale reflects the values of phase fluctuations in radians. It is worth noting that there are several models used to generate turbulent phase screens. Currently, the most common methods for turbulent phase screen generation use an FFT of random spectral density with statistics matching the phase turbulence to a certain extent. The FFT phase is complemented by subharmonic terms that compensate for the lack of low-frequency FFT components. However, it is only capable of generating phase screens that are restricted to a rectangular spatial grid [44]. The validity of the turbulent phase screen model generated by the FFT method has been proven in our previous studies [42].

## 3. Results and Discussion

In our numerical simulation, we set topological charge *l* as 10, since it has been reported that a large topological charge can reduce the influence of air turbulence during propagation [45]. Recently, the axicon has become a promising optical device in elongating the length of a single filament [41,46,47]. Therefore, a vortex beam with a topological charge of 10, which propagates through a lens or an axicon, has been considered, respectively, during our work.

**Figure 2:**
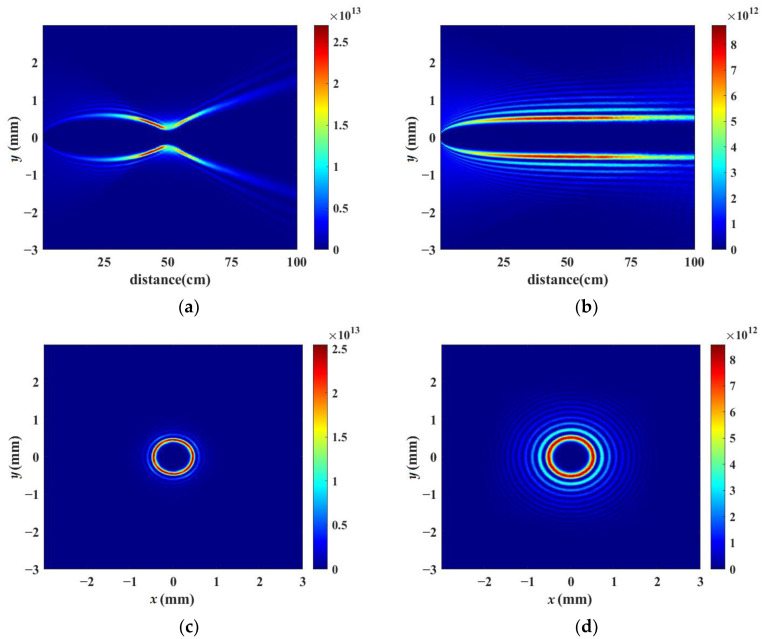
Simulated intensity distribution without air turbulence by using (**a**) a lens and (**b**) an axicon longitudinal distribution during nonlinear propagation when the ultrafast laser powers are set to 20 times the critical power, respectively. (**c**,**d**) transverse distribution at *z* = 60 cm during nonlinear propagation corresponding to (**a**,**b**).

Figure 2a,b demonstrate the simulated vortex beam’s intensity distribution along the axis of *x* = 0 during nonlinear propagation when the laser powers are set as 20 times the critical power for self-focusing by using a lens and an axicon without air turbulence, respectively. Figure 2a shows that the vortex beam is focused by the lens of f = 50 cm, which corresponds to one half of the whole propagation distance, 100 cm. Curved filamentation is formed by the focusing lens, which can be explained by the strong energy confinement along a bent trajectory caused by vortex phase modulation introduced into the initial beam [48]. Figure 2c,d indicate the corresponding cross-section patterns of laser intensity at the propagation distance *z* = 60 cm. There are some ring structures shown in Figure 2c,d. Figure 2b,d shows that after being focused by an axicon, a vortex beam can be converted into a high-order Bessel beam. Moreover, the order of the Bessel beam is equal to the topological charge number of the vortex beam. The bottom angle of the axicon was set as 0.28° in the simulation, so that the non-diffraction zone of the axicon coincided with the propagation distance of 100 cm.

In order to perform the statistical regularities of vortex beam propagation in turbulent air, we used 20 independent simulations with different series of stochastic turbulence phase screens for each condition. The turbulent phase screens were introduced at the positions of *z* = 0 cm, 25 cm, 50 cm and 75 cm, corresponding to the beginning position of the initial vortex beam, the onset, the intermediate stage and the end of the filamentation process, respectively. The receiving screen was placed at the location of 100 cm to record the transverse intensity patterns during the propagation process of the laser beams.

**Figure 3:**
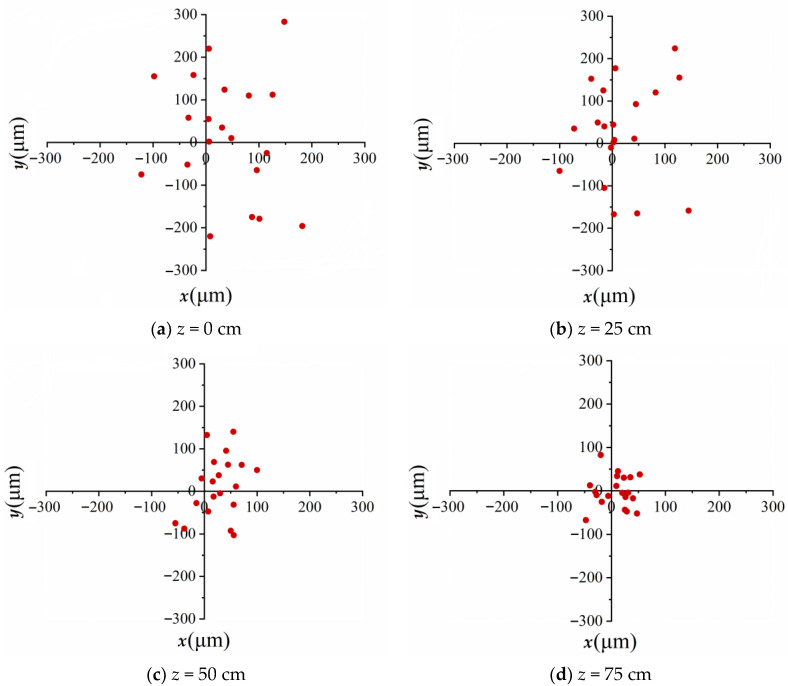
Spatial displacements focused by a lens (f = 50 cm) when the air turbulence (C_n_^2^ = 5.6 × 10^−13^ cm^−2/3^) operates at *z* = (**a**) 0 cm, (**b**) 25 cm, (**c**) 50 cm and (**d**) 75 cm during nonlinear propagation.

Figure 3 demonstrates the simulated results of the statistical characteristics of vortex beam wandering focused by a lens when the air turbulence with the structure constant C_n_^2^ = 5.6 × 10^−13^ cm^−2/3^ operates in the nonlinear propagation for every 25 cm of distance, respectively. Because of the ring structure of the transverse intensity distribution, as opposed to a single spot, the central coordinates (*x*_0_, *y*_0_) of the vortex beam were calculated with the following formula:

(8) $$x_{0}=\frac{\iint xI(x,y)dxdy}{\iint I(x,y)dxdy},y_{0}=\frac{\iint yI(x,y)dxdy}{\iint I(x,y)dxdy}$$

where *I* (*x*, *y*) refers to the ring structure intensity generated by the vortex beam. Figure 3 implies that the resulting vortex beam wandering was random due to the stochastic characteristic of turbulence. The red dots correspond to the central coordinates (*x*_0_, *y*_0_) of vortex beams. The simulations have been repeated 20 times for each position where the turbulent phase screens were introduced; thus, there are 20 red dots in each of Figure 3a–d. The average values of the transverse intensity central displacement <*R*_0_>, which is defined as $R_{0}=\sqrt{x_{0}^{2}+y_{0}^{2}}$, are approximately 128 μm, 101 μm, 71 μm and 40 μm, corresponding to the locations of 0 cm, 25 cm, 50 cm and 75 cm, where the turbulent phase screens were placed. The turbulence phase screens will lead to phase perturbation and provoke the instability of the wavefront continuously; thus, the influence of turbulent air on beam wandering is stronger when the turbulence occurs prior to the onset of propagation as compared to when it takes place at the middle or the end of propagation. Therefore, we can conclude that the air turbulence occurring earlier can result in a greater influence on the beam wandering. In the meantime, we numerically simulated a vortex beam propagating through a lens in turbulent air using 20 different series of stochastic turbulence phase screens during linear propagation. The average values <*R*_0_> calculated were 141 μm, 119 μm, 91 μm and 48 μm, when the phase screens were placed at 0 cm, 25 cm, 50 cm and 75 cm, respectively. The average values of spatial displacement become 110%, 118%, 128% and 120% of that during nonlinear propagation at the same location. It is well established that the turbulence effect’s contribution to the refractive index gradient is negligible compared with the nonlinear effect, which was induced by the Kerr effect within the filamentation. Previous work reported that filamentation was very resistant to turbulence once it was formed [34]. Thus, we included the consideration that the nonlinear evolution of the vortex beam can suppress the air turbulence better than linear evolution.

**Figure 4:**
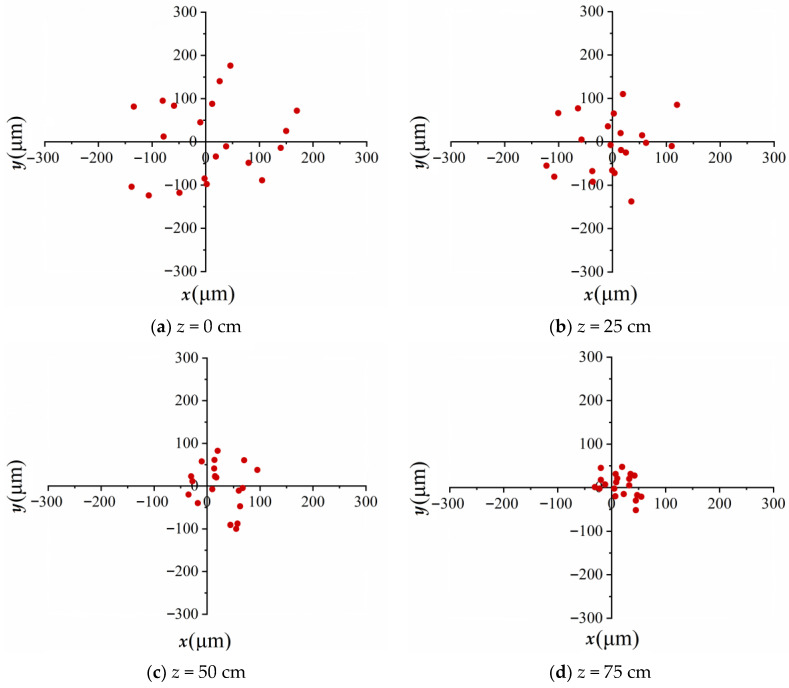
Spatial displacements focused by an axicon (the bottom angle is 0.28°) when the air turbulence (C*_n_*^2^ = 5.6 × 10^−13^ cm^−2/3^) operates at *z* = (**a**) 0 cm, (**b**) 25 cm, (**c**) 50 cm and (**d**) 75 cm during nonlinear propagation.

As a comparison, Figure 4 illustrates the simulated results of vortex beam wandering focused by an axicon when the air turbulence with the structure constant C_n_^2^ = 5.6 × 10^−13^ cm^−2/3^ operates at different positions of 0 cm, 25 cm, 50 cm and 75 cm during nonlinear propagation. The numerically transverse intensity patterns were recorded at 100 cm. The average values of the transverse intensity central displacement <*R*_0_> are approximately 101 μm, 74 μm, 55μm and 33 μm, which become 79%, 73%, 77% and 78% of that focused by a lens at the same location. Using the same methods mentioned above, vortex beam wandering focused by an axicon was simulated in linear propagation. The average values *<R*_0_*>* calculated are 109 μm, 89 μm, 65 μm and 36 μm, which are all approximately 75% more or less than that focused by a lens during linear propagation. Because of the non-diffraction propagation, it is indicated that the axicon, as the focusing optical device, can suppress the optical perturbation induced by air turbulence.

The angle-of-arrival (AOA) fluctuation plays a significant role in FSO communication and atmospheric remote sensing [49]. The AOA fluctuation of an optical wavefront of the receiver aperture is associated with distortion in the focal plane of an imaging system. Under the geometrical optics method, the definition of AOA has the following form [50]:

(9) $$\beta =\arctan\frac{\Delta S}{f^{\prime }}\underset{}{}(radian),$$

where Δ*S* represents the phase shift, and *f*’ is the focal length of the receiving antenna; we set *f*’ = 1 m for convenience in the simulations. The AOA fluctuation is defined as the standard deviation of *β*.

**Figure 5:**
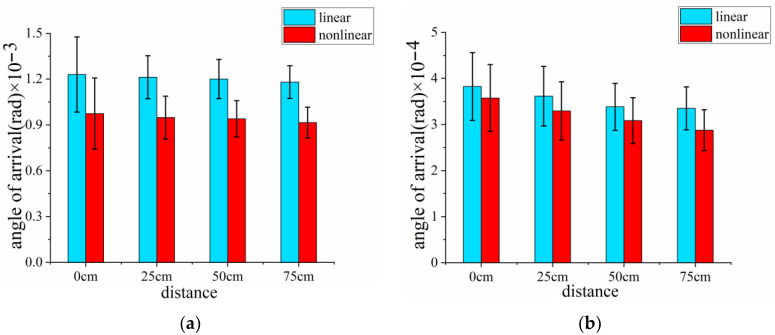
The simulated average values of AOA of vortex beams focused by (**a**) a lens and (**b**) an axicon when turbulence was introduced at various displacements, respectively, during linear (blue) and nonlinear (red) propagation.

Figure 5 shows the simulated average values of AOA of vortex beams focused by a lens and an axicon when air turbulence was introduced at the various positions (*z* = 0, 25, 50 and 75 cm), respectively, during linear and nonlinear propagation. The error bar of Figure 5 denotes the AOA fluctuation. We can see that the average values of AOA decreased gradually when the air turbulence screen was placed from the beginning to the end of the propagation. Meanwhile, it can be observed that there are no obvious differences among the various positions where turbulent screens were installed. Therefore, the AOA fluctuation is not significantly affected by the different stages of propagation compared with the results of beam wandering as shown in Figure 3 and Figure 4. Meanwhile, the average values of AOA in nonlinear propagation are slightly smaller than that in the linear condition. As a comparison, we discovered, surprisingly, that the average values of AOA of the vortex beam focused by an axicon are nearly an order of magnitude lower than that focused by a lens. The results further confirm that a laser beam propagating through an axicon with a relatively large bottom angle (generally ≥ 0.2°) not only can suppress the turbulence, but can also maintain a more stable beam pattern.

In addition, we discussed the degree of transverse intensity patterns’ distortion. Figure 2b,d illustrate that the ring structure created by the vortex beam is usually of a circular pattern. However, due to the optical perturbation in the atmosphere, the circular-shaped ring structure is easily transformed into an elliptic or other irregular oval shape. Here, distortion factor *δ* is expressed by the following formula:

(10) $$\delta =\frac{\Delta a}{\Delta b},$$

where Δa is the maximum distance between the edges of the ring structure along the *x*-axis and Δb is along the *y*-axis.

**Figure 6:**
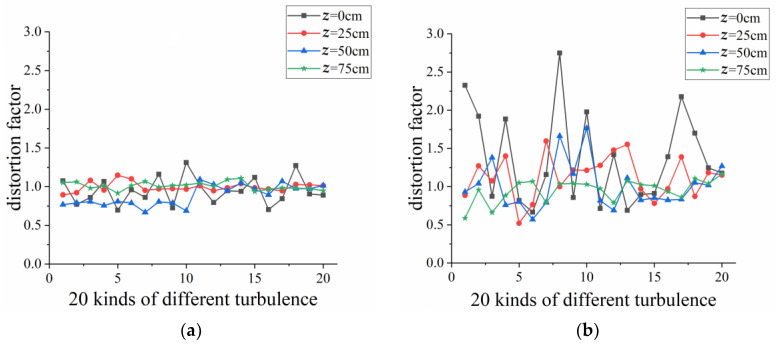
The distortion factor *δ* of the ring structure generated by (**a**) a lens and (**b**) an axicon during nonlinear propagation with 20 different turbulence screens located at various positions, respectively.

Figure 6 depicts the distortion factor *δ* of the ring structure generated by a lens and an axicon during nonlinear propagation when 20 different turbulence screens were located at *z* = 0 cm, 25 cm, 50 cm, 75 cm, respectively. Figure 6 shows that the variation in distortion factors focused by an axicon are more random and irregular than that focused by a lens during nonlinear propagation. It can be interpreted that the ring transverse intensity of vortex beams focused by a lens is usually larger than that focused by an axicon due to the divergence; therefore, small changes in Δ*a* and Δ*b* can lead to great fluctuations in distortion factor *δ* according to Equation (10).

**Figure 7:**
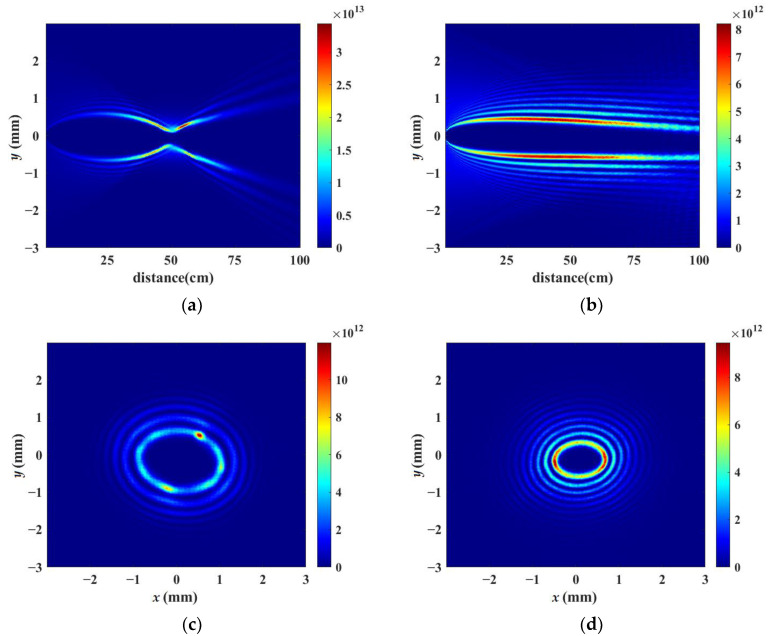
Representative simulated intensity distribution with 4 turbulent phase screens in the whole propagation process by using (**a**) a lens and (**b**) an axicon, showing longitudinal distribution during nonlinear propagation, respectively; (**c**,**d**) transverse distribution at *z* = 70 cm during nonlinear propagation corresponding to (**a**,**b**).

In order to simulate the real effect of air turbulence in the atmosphere, the first turbulent phase screen was placed at the beginning of the propagation of the initial vortex beam and another three phase screens of the same series were introduced along the propagation path at every 25 cm simultaneously. The receiving screen was placed at the location of 100 cm to record the transverse intensity patterns. In other words, in each numerical simulation, a total of four phase screens were set up along the entire propagation path to fit the actual atmospheric conditions. The simulations have been repeated 20 times using a different series of air turbulence screens with the same structure constant of C_n_^2^ = 5.6 × 10^−13^ cm^−2/3^. Figure 7 demonstrates the representative simulated vortex beam intensity distribution focused by a lens and an axicon with four turbulent screens simultaneously located at four different positions during the whole nonlinear propagation process when the laser powers are set as 20 times the critical power for self-focusing. Figure 7a,b depict the vortex beam intensity distribution along the axis of *x* = 0, while Figure 7c,d indicate the corresponding cross-section patterns of laser intensity at the propagation distance *z* = 70 cm. Compared with Figure 2, the vortex beam intensity distribution does not show cylindrical symmetry due to the effect of air turbulence. There are several “hot” spots along the ring structure shown in Figure 7c, which may lead to several single filaments. Meanwhile, it can be observed in Figure 7b that the high-order Bessel beam generated by the vortex beam propagating across the axicon does not transmit in a straight manner along the central axis. As shown in Figure 7d, the transverse intensity distribution of the Bessel beam is converted into an inhomogeneous elliptical pattern.

**Figure 8:**
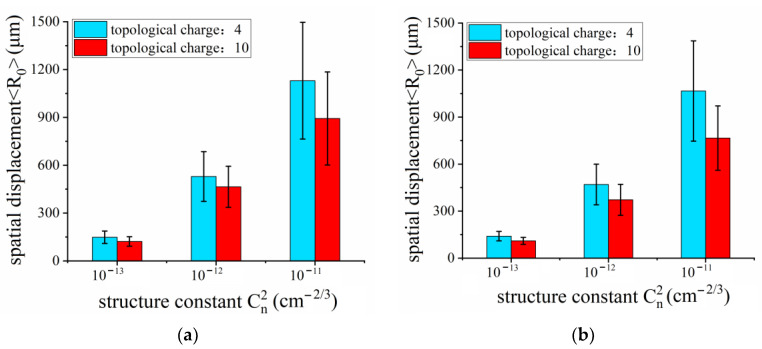
The average spatial displacements <*R*_0_> as a function of turbulent structure constant *C_n_^2^* when vortex beams carrying 4 (blue) and 10 (red) modes of OAM state propagate through (**a**) a lens and (**b**) an axicon during nonlinear propagation.

Although there are some other parameters in atmospheric air, such as temperature, relative humidity and wind speed, these effects eventually lead to the variation in turbulent structure constant C_n_^2^ [51]. The optical effects of atmospheric turbulence depend primarily on the structure parameter C_n_^2^. Consequently, we performed a statistical analysis of the transverse spatial displacements as a function of turbulent structure constant C_n_^2^. As shown in Figure 8, by varying structure constant C_n_^2^ from 10^−13^ to 10^−11^ cm^−2/3^, we simulated the nonlinear propagation process of a vortex beam focused by a lens and an axicon with four turbulent screens simultaneously. In the meantime, we set the topological charge as 4 in addition to 10 to investigate the properties of another mode of OAM state carried by vortex beam propagation in turbulent air. Each process of simulation was repeated 20 times with a different series of air turbulence screens. Figure 8 describes the average spatial displacements <*R*_0_> as a function of turbulent structure constant C_n_^2^ when vortex beams carrying 4 and 10 modes of OAM state propagate through a lens and an axicon during nonlinear propagation (P = 20P_cr_). The average values of spatial displacements <*R*_0_> become gradually larger with the increasing structure constant C_n_^2^. According to Equations (1)–(7), it can be easily understood that the turbulent structure constant contributes to the refractive index gradient more efficiently than any other parameter. Thus, the larger the structure constant is, the more intensively the air turbulence affects the beam wandering. Comparing the topological charge of 10 with that of 4 in Figure 8, the relatively larger topological charge behaves better with respect to suppressing the effect of air turbulence, which is consistent with the previously reported conclusion [45].

## 4. Conclusions

In summary, we have investigated the statistical regularities of a vortex beam propagating through a lens or an axicon with different series of turbulent air phase screens. We mainly simulated the evolution process of beam wandering owing to turbulent phase screens with various parameters. The results revealed that the distortion of the spatial displacement was sensitive to the location and the structure constant C_n_^2^ of the turbulent screens. The effect of the optical perturbation owing to air turbulence is more intensive when the phase screen is located at the early stages of the propagation. Turbulent structure constant C_n_^2^, which can affect the refractive index gradient efficiently, is a significant parameter in the atmospheric air. Compared with linear propagation, we find that the nonlinear process of vortex beams can effectively suppress the effect of air turbulence. In addition, the axicon can be regarded as a very useful optical device, since it can not only suppress the turbulence but also maintain a stable beam pattern. Furthermore, we simulated vortex beams carrying different OAM modes propagating in turbulent air. It can be concluded that a large topological charge can reduce the influence of air turbulence during propagation. Our outcomes are valuable for many applications in atmospheric air, especially for optical communication and remote sensing.

## Data Availability

The data presented in this study are available on request from the corresponding author.
